# Self-Esteem and Coping Strategies in Adolescent Cancer Patients during the Period of Illness and Follow-Up

**DOI:** 10.3390/ejihpe14050074

**Published:** 2024-04-24

**Authors:** Diego José Sáez Rodríguez, Juan Manuel Ortigosa Quiles, Antonio Riquelme Marin, Raquel Suriá Martínez

**Affiliations:** 1 Department of Personality, Assessment and Psychological Treatments, Faculty of Psychology, University of Murcia, Espinardo University Campus, 30100 Murcia, Spain; ortigosa@um.es (J.M.O.Q.); riquelme@um.es (A.R.M.); 2 Department of Communication and Social Psychology, University Institute for Gender Studies Research, University of Alicante, Social Sciences Building, 03690 Alicante, Spain; raquel.suria@ua.es

**Keywords:** self-esteem, personal resources, cancer, coping, illness period, adolescence

## Abstract

The importance of self-esteem during the course of oncological illness has been well-documented by some previous studies. However, data assessing its association with various coping strategies, especially considering the period of illness, are still scarce. The objective of this study is to analyze the differences in coping strategies among oncological adolescents, taking into account their self-esteem, illness period, age, and sex. A total of 201 oncological patients between the ages of 12 and 17 from three different Spanish cities were included in this study. All of them were asked to answer a tailored questionnaire, encompassing information about age, sex, and illness period. Additionally, the coping strategies were measured using the ACS scale, while self-esteem was evaluated using the SENA questionnaire. The results demonstrated that male adolescents and older individuals exhibited higher levels of self-esteem. The main coping strategies associated with higher self-esteem were “ignore the problem”, “focus on positive”, “physical recreation”, and “wishful thinking” both during the treatment and the follow-up phases. We conclude that higher self-esteem is associated with some of the coping strategies such as “focus on positive”, “ignore the problem”, and “wishful thinking”. Sociodemographic variables influence the relationship between self-esteem and coping strategies, but no differences were found regarding the period of illness.

## 1. Introduction

Oncological diseases are considered chronic disorders with multifactorial etiology, affecting individuals across all age groups including adults, children, and adolescents [1]. However, due to the low detection rate of cancer in children and adolescents [2], along with the significant demand for resources and optimized interventions [3], prevention becomes increasingly challenging within these age groups [4].

According to the Spanish Association against Cancer, a total of 951 male adolescents and female adolescents between zero and 14 years old were diagnosed with cancer in Spain during 2022. Additionally, the previous year’s data indicated a mortality rate of two per 100,000 inhabitants [5].

The epidemiological impact is associated with the risk of experiencing certain psychological difficulties such as depression and anxiety, particularly among adolescents dealing with long-term physical restrictions [6], in whom a series of psychological and social disruptions may occur, including vulnerability, panic, and isolation, affecting both the families and the patients themselves [7].

Children and adolescents with cancer express how challenging it is to face the changes and fears they experience. Nevertheless, they attribute a meaning of transformation and personal growth to the disease, while also accepting negative thoughts such as the fear of death or uncertainty [8].

Aspects like self-esteem improve when psychological interventions are carried out during the oncological treatment, such as psychoeducation, emotional support, and coping strategies [9,10]. Additionally, there are variations between sexes in terms of coping strategies and self-esteem levels [11].

Cancer is related to self-esteem [12], generating a sense of lack of control during a crucial period for healthy development when self-perception and achieving independence are essential [13]. The transition to adulthood is often disrupted when individuals cope with both the regular demand of development and the challenges associated with the diagnosis, thereby making it more challenging to engage in self-exploration and autonomy gain [14].

Precise knowledge about the effects of a cancer diagnosis during adolescence remains scarce [15]. A life-threatening illness during this vulnerable stage of development can lead to more significant psychosocial consequences compared to cancer experienced during childhood [16]. This is because the disease increases the need for dependence on caregivers and reduces participation in activities with peers of the same age [17], making it difficult to accomplish the numerous typical developmental tasks associated with adolescence [18].

Due to the emotional impact of cancer on children and adolescents, studies such as those by Hensel et al. [19] and Erickson and Steiner [20] have primarily focused on the psychological pathology of the patient. In contrast, other works like those of Hullmann et al. [21] and Barakat et al. [22] have revealed a series of more adaptive factors, such as quality of life, hope, optimism, and coping strategies. It has been observed in both patients and parents that this illness has also led to an increase in psychological resilience and the ability to confront the consequences of this disease [23].

Adolescents perceive the likelihood of relapse to be higher and, as a result, healthcare professionals encourage them to use fewer avoidance-focused coping strategies. Instead, they recommend coping strategies that involve acceptance and problem resolution [24]. Despite the potential negative connotations associated with the probability of relapse and taking into account the positive perspective discussed earlier, the coping strategy of acceptance is reinforced, leading to enhanced personal growth following the experience of the disease [25].

Throughout the course of their illnesses, adolescents acquire valuable skills in stress management, goal setting, and seeking benefits, all of which empower them throughout their experiences with cancer, leading to improved long-term psychosocial outcomes [26]. Additionally, there is a need for young individuals to strive to regain the state they were in before falling ill [12]. Regardless of the stage of illness they are in, adolescents undergoing an oncological process tend to employ similar coping strategies [27].

Despite these positive aspects, the illness gives rise to several adverse effects. The most common side effect during oncological processes in children and adolescents is low self-esteem, as compared to the general population, primarily due to their stage of development [28]. This phenomenon is considered highly relevant during this period as it plays a pivotal role in fostering an individual’s sense of security, positive identity, and self-worth. This attribute can serve as an indicator of their readiness for psychological or emotional personal changes [29]. Furthermore, it is established that high self-esteem leads to an increased utilization of various coping strategies in both adolescents [30] and oncological women [31]. In addition to these findings, increased self-esteem is also associated with improvement in the psychological well-beings of adolescents [32].

By employing effective coping strategies, individuals achieve higher levels of psychological well-being and adapt better to challenging situations. These strategies enable them to regulate emotions and find suitable solutions to the difficulties they face, promoting better psychological adjustment and increased resilience [33]. Moreover, they play an important role in developing positive coping skills and reducing emotion-focused approaches, which can directly influence emotional aspects, including self-esteem [34].

Many adolescents perceive the experience of illness as a period of transition and therefore rely on external resources such as family support, healthcare professionals, and friendships at school. Additionally, they also draw upon internal resources, such as religion, resilience, and other personal strengths [35].

The coping strategies “focus on positive”, “physical recreation”, and “work hard” are associated with greater personal well-being, while strategies like “self-blame” and “keep to myself” lead to a decrease in well-being [36]. Similarly, avoidance and denial strategies are linked to higher psychological symptoms [37] as well as to a decrease in quality of life, and self-esteem and an increase in psychological distress [38].

Given the aforementioned factors, it is essential to analyze the relationship between self-esteem and coping strategies in adolescents. Additionally, there is a need to study these variables during different phases of the illness, such as the treatment period when the patient is undergoing hospitalization and treatment or the post-treatment or follow-up period, considering that the distinction between these phases impacts individuals’ personal resources and social contexts [39].

Hence, the aim of this study is to evaluate the coping differences in adolescent oncology patients in relation to their self-esteem as a personal resource, considering the periods of illness, age, and sex of the participants. Additionally, it is hypothesized that there will be a relation between the use of various coping strategies and the patients’ levels of self-esteem, age, and sex. Similarly, patients in the follow-up period will differ in their utilization of various coping strategies compared to those in the illness period.

## 2. Methods

### 2.1. Design

Quantitative and descriptive study was used to employ an observational cross-sectional methodology.

### 2.2. Participants

The sample was selected through the associations Aspanion and Asion. Aspanion granted access to patients from the General University Hospital Dr. Balmis and the University and Polytechnic Hospital La Fe. Simultaneously, access to patients from the University Childrens’ Hospital Niño Jesús was approved by the association Asion. Inclusion criteria for the study involved patients aged 12 to 17 years who had received an oncological diagnosis.

Contact was established via telephone calls, using information provided by the associations and adhering to legal confidentiality terms. During the phone call, the research objectives and execution plan were explained to the parents. Once families agreed to participate, the questionnaire was administered.

The study involved 201 users of any of the association’s programs (social support, psychological, neuropsychological, leisure and free time program, volunteering, etc.), accessible from the hospital setting and through the support provided by these associations.

### 2.3. Instruments

The final model of the structured interview comprised the following instruments:

(a) Ad hoc questionnaire, where data regarding age, sex, and the period of illness were collected, distinguishing between the treatment and follow-up periods.
(b) Adolescent Coping Scale (ACS) by Frydenberg and Lewis: This instrument, adapted for the Spanish population, evaluates 18 coping strategies including “social support”, “solving problem”, “work hard”, “worry”, “friends”, “belong”, “wishful thinking”, “not coping”, “tension reduction”, “social action”, “ignore the problem”, “self-blame”, “keep to myself”, “spiritual support”, “focus on positive”, “professional help”, “relaxing diversions”, and “physical recreation”. The test was tailored so that respondents could provide both non-specific responses (about how they cope with their problems in general) and specific responses (how they deal with a particular problem that especially concerns them or that the counselor, tutor, or clinician wants to address) [40].
(c) Children and Adolescents Assessment System (SENA) by Fernández-Pinto, Santamaría, Sánchez-Sánchez, Carrasco, and del Barrio: an instrument aimed at detecting a wide range of emotional and behavioral problems in individuals aged three to 18 years. It assesses: (1) internalized problems: depression, anxiety, social anxiety, somatic complaints, obsessive-compulsive symptoms, and post-traumatic symptomatology and (2) externalized problems: hyperactivity and impulsivity, attention disorders, aggressiveness, defiant behavior, anger control disorders, and antisocial behavior. Additionally, other variables were evaluated including self-esteem. Information was collected from both the adolescents themselves and their families [29,30].

### 2.4. Procedure

The research was approved by the Ethics Committee of the University of Murcia (CEI 2870/2020), followed by the Ethics Committees of the General University Hospital Dr. Balmis and the University and Polytechnic Hospital La Fe, in collaboration with Aspanion and the Ethics Committee of the University Childrens’ Hospital Niño Jesús, in partnership with Asion. Once approved, access to the study sample was granted through a duly complimented authorization form, containing all the research details, so that parents or guardians could provide consent, allowing us to directly intervene with the sample.

First, informed consent was obtained from the study participants themselves. Next, the intervention was structured as a set of self-administered questionnaires conducted online, with the subject receiving assistance from the interviewer, making the intervention as non-invasive as possible. This procedure also allowed patients to complete the information when they were capable of collaborating with the project.

### 2.5. Data Analysis

First, a descriptive analysis was conducted for the sociodemographic variables of age, sex, and period of illness, using absolute and relative frequencies in percentages. An analysis of covariance was performed to examine the relationship between age, sex, period of illness, and self-esteem. Additionally, the interaction between sex and period of illness concerning self-esteem was studied. Subsequently, to analyze the association between self-esteem as a personal resource and the 18 coping strategies, a correlation matrix was created, and linear regression models were employed to assess the relationships between coping strategies and self-esteem, taking into account sex and period of illness. The data analysis was performed using the statistical package IBM^®^ SPSS Statistics v.26.0 with a significance level set at *p* < 0.05.

## 3. Results

Based on the period of illness, 37 male adolescents (41.1%) and 53 female adolescents (58.9%) were undergoing treatment, while 45 male adolescents (40.5%) and 66 female adolescents (59.5%) were in a state of discharge or follow-up.

The data revealed a significant association between self-esteem and age (F = 18.7, *p* < 0.001) and sex (F = 11.23, *p* = 0.001). However, no association was found between self-esteem and the period of illness (F = 0.09, *p* = 0.764), as shown in Table 1.

The mean self-esteem score for male adolescents was 56.06, and for female adolescents it was 53.87. The rest of the mean scores based on the period of illness and sex are presented in Table 2.

A positive correlation was observed between age and self-esteem (rxy = 0.206, *p* < 0.001), as depicted in Table 3.

A regression analysis was conducted to explore the association between self-esteem and coping strategies. As shown in Table 4, the coping strategies “physical recreation”, “ignore the problem”, “worry”, “belong”, and “social action” jointly explained 63.5% of self-esteem.

Next, separate regression models were defined for male adolescents and female adolescents, examining the relationship between self-esteem and coping strategies. For male adolescents, as displayed in Table 5, the strategies “physical recreation” and “focus on positive” explained 81.7% of the variance in self-esteem. For female adolescents, as presented in Table 6, the strategies “focus on positive”, “solving problem”, “relaxing diversions”, “ignore the problem”, “wishful thinking”, and “spiritual support” explained 80.7% of self-esteem.

A general regression model was also established between self-esteem and coping strategies during the treatment period. As shown in Table 7, the strategies “focus on positive”, “ignore the problem”, “wishful thinking”, “physical recreation”, and “worry” collectively explained 58.6% of self-esteem.

After the analysis of the treatment period, a general regression model was established for the follow-up period to assess the relationship between self-esteem and coping strategies. As indicated in Table 8, the strategies “focus on positive”, “ignore the problem”, “wishful thinking”, and “spiritual support” explained 56.6% of self-esteem.

In line with the previous models, a regression analysis was performed separately for male adolescents and female adolescents to evaluate the relationship between self-esteem and coping strategies during the treatment period. As shown in Table 9, for male adolescents, the strategies “keep to myself”, “work hard”, “solving problem”, and “belong” collectively explained 79.2% of self-esteem. Similarly, for female adolescents, as displayed in Table 10, the strategies “focus on positive”, “ignore the problem”, “wishful thinking”, “spiritual support”, and “friends” explained 83.8% of self-esteem.

Finally, separate regression models were created for male adolescents and female adolescents during the follow-up period to evaluate the relationship between self-esteem and coping strategies. For male adolescents, as presented in Table 11, the strategies “not coping”, “belong”, “keep to myself”, and “ignore the problem” collectively explained 80.2% of self-esteem. In the case of female adolescents, as indicated in Table 12, the strategies “focus on positive”, “physical recreation”, “ignore the problem”, “keep to myself”, and “professional help” jointly accounted for 84.6% of self-esteem.

## 4. Discussion and Conclusions

The aim of this study was to analyze the coping differences among adolescent oncology patients in relation to self-esteem as a personal resource, considering the period of illness, age, and sex of the participants. The obtained results indicate that both sex and age are significantly associated with the perception of self-esteem in the included patients, with higher levels observed in male adolescents and older individuals. Moreover, the findings highlight the relationship between self-esteem and the general coping strategies used by adolescent oncology patients and are consistent with the findings of Huapaya [30]. In particular, the strategies of “physical recreation”, “ignore the problem”, “worry”, “belong”, and “social action” show the strongest association with self-esteem, confirming the findings of Cheng et al. [33], McMahon et al. [34], and Cadena et al. [35].

Thus, the hypothesis of an association between coping strategies and self-esteem is partially supported in this study. Specifically, a positive correlation was found between the coping strategy of “physical recreation” and self-esteem, while it was not significant between self-esteem and the coping strategies of “focus on positive” and “work hard”.

These results align with previous findings by Poch et al. [36], which also demonstrated a similar trend of association. However, our study does not replicate the findings of Compas et al. [37], Sepúlveda and Carrillo [39], and Turner-Sack et al. [24] regarding a decrease in self-esteem in relation to avoidance and denial strategies.

Regarding age, the hypothesis is rejected as the results obtained demonstrate a direct and proportional increase in self-esteem within the studied sample. However, concerning sex, the hypothesis is supported, as female adolescents utilize a wider range of coping strategies compared to male adolescents. While many studies do not specify age or sex differences, our research highlights these distinctions, which could have significant implications for future studies. This finding aligns with Mittmann and Schrank [11], where variability in coping strategies and self-esteem based on sex was reported.

Furthermore, higher levels of self-esteem in adolescents are associated with greater overall usage of coping strategies. These findings are consistent with those reported by Huapaya [30], Wang et al. [9], and Azem [10], suggesting that interventions that focus on coping strategies can lead to increased self-esteem. Consequently, it becomes important to encourage the use of effective coping strategies among adolescents in this context to promote higher levels of self-esteem.

Finally, and in relation with Sepúlveda and Carrillo [39], the results regarding the treatment period could initially suggest differences between the illness and follow-up periods due to variations in social, educational, and family contexts among others, which confirms the findings of García-García and Lucio [27] that the hypothesis that patients in these different periods differ in the use of coping strategies is rejected. Instead, a significant similarity is observed in the coping strategies used during both periods, presenting one of the novel conclusions of this study, as the scientific literature has not previously explored the existence of such differences.

Regarding the study’s limitations, the recruitment of new participants was not possible due to the COVID-19 health emergency. Replicating the study with a larger sample would be interesting for observing differences based on the type of illness, academic year, and other sociodemographic variables that may influence the level of self-esteem in relation to coping strategies. Despite this limitation, the results are valuable for direct intervention on personal resources, potentially enhancing the appropriate use of coping styles.

Nevertheless, it is essential to continue refining the available tools to improve these personal resources and significantly implement the use of coping strategies in oncological processes, in line with the studies presented by Hullmann et al. [21] and Barakat et al. [22].

These effects carry important practical implications, as they suggest that individuals with self-esteem issues could benefit from learning and applying more effective coping strategies. For instance, promoting seeking social support or engaging in social activities as coping mechanisms may prove useful in improving the self-esteem of these patients.

These findings have the potential to guide the design of more personalized and effective interventions adapted to the changing demands throughout the course of the disease and taking into account age, sex, and the period of illness of each patient. 

From the clinical point of view, the use of more internal resources should be promoted in patients with lower levels of self-esteem, in addition to trying to avoid avoidance strategies in order to achieve internal perceptions of their diseases and thus intensify the feelings of integration and usefulness within the disease processes. On the other hand, in patients with high levels of self-esteem, it is relevant to promote aspects of sociability and to support the search for external help, if necessary, in order to optimize their individual identity during these procedures. 

In the fields of mental and medical health, orientation towards adaptive strategies could be integrated into therapeutic programs to strengthen the overall well-being of these individuals. At the same time, addressing less healthy strategies could be the key to promoting more positive and functional adjustments in coping with adversity. 

Health professionals should consider natural adjustment in coping strategies throughout the life cycle, adapting therapeutic approaches that value reflection on goals and acceptance of physical limitations at different stages of life. Likewise, there is a need to address changes in social networks as age increases in order to encourage more meaningful and selective interactions to optimize social support. Transformations in beliefs and leisure preferences over time should also be taken into consideration to adapt interventions that respect and align with individual evolution.

In summary, this study indicates that the coping strategies employed by young individuals can significantly impact their self-esteem, and these factors may differ between sexes. Therefore, sex perspective must be taken into account in the development of effective interventions to enhance the self-esteem of young individuals.

This study emphasizes that incorporating these coping strategies into intervention programs to enhance self-esteem is important and can significantly improve therapeutic outcomes. Promoting the use of strategies such as “focus on positive”, “ignore the problem”, and “wishful thinking” could serve as intervention targets to enhance self-esteem. Furthermore, these results highlight the importance of evaluating and reevaluating the relationship between coping strategies and self-esteem during the design of interventions for these patients.

## Figures and Tables

**Table 1:** ANCOVA between age, sex, and period of illness on self-esteem.

Source	Type III Sum of Squares	df	F	*p*	Partial Eta Squared
Corrected model	1064.434 ^a^	4	6.561	<0.001	0.118
Intercept	4450.713	1	109.727	<0.001	0.359
Age	758.507	1	18.7	<0.001	0.087
Sex	455.402	1	11.227	0.001	0.054
Period of illness	3.653	1	0.09	0.764	0.000
Sex x Period of illness	61.856	1	1.525	0.218	0.008
Error	7950.104	196	6.561		
Total	611,771	201	109.727		
Corrected total	9014.537	200	18.7		

^a^ R squared = 0.118 (adjusted R squared = 0.100).

**Table 2:** Analysis of self-esteem according to sex and period of illness.

Sex	Period of Illness	Mean	Standard Deviation	N
Male	Illness period	56.86	4.632	37
	Follow-up period	55.40	5.466	45
	Total	56.06	5.129	82
Female	Illness period	53.32	8.057	53
	Follow-up period	54.30	7.067	66
	Total	53.87	7.507	119
Total	Illness period	54.78	7.048	90
	Follow-up period	54.75	6.462	111
	Total	54.76	6.714	201

**Table 3:** Correlations between self-esteem and age.

		Age	Self-Esteem
Sex	Pearson Correlation	0.206 **	−0.161 *
	Sig. (2-tailed)	0.003	0.022
	N	201	201
Age	Pearson Correlation	1	0.252 **
	Sig. (2-tailed)		0.000
	N	201	201
Self-esteem	Pearson Correlation	0.252 **	1
	Sig. (2-tailed)	0.000	
	N	201	201

** Correlation is significant at the 0.01 level (2-tailed). * Correlation is significant at the 0.05 level (2-tailed).

**Table 4:** Regression model between self-esteem and different coping strategies.

Model	R	R Square	R Square Change	Cumulative R Square	Sig.
1	0.489	0.239	0.239	0.239	<0.001
2	0.655	0.430	0.190	0.429	<0.001
3	0.721	0.520	0.091	0.520	<0.001
4	0.768	0.589	0.069	0.589	<0.001
5	0.797	0.635	0.046	0.635	<0.001

1. Predictors: Physical recreation. 2. Predictors: Physical recreation and ignore the problem. 3. Predictors: Physical recreation, ignore the problem, and worry. 4. Predictors: Physical recreation, ignore the problem, worry, and belong. 5. Predictors: Physical recreation, ignore the problem, worry, belong, and social action.

**Table 5:** Regression model between self-esteem and different coping strategies in male adolescents.

Model	R	R Square	R Square Change	Cumulative R Square	Sig.
1	0.582	0.338	0.338	0.338	<0.001
2	0.904	0.817	0.479	0.817	<0.001

1. Predictors: Physical recreation. 2. Predictors: Physical recreation and focus on positive.

**Table 6:** Regression model between self-esteem and different coping strategies in female adolescents.

Model	R	R Square	R Square Change	Cumulative R Square	Sig.
1	0.679	0.461	0.461	0.461	<0.001
2	0.742	0.550	0.089	0.550	<0.001
3	0.797	0.634	0.084	0.634	<0.001
4	0.836	0.699	0.065	0.699	<0.001
5	0.875	0.765	0.066	0.765	<0.001
6	0.899	0.807	0.042	0.807	<0.001

1. Predictors: Focus on positive. 2. Predictors: Focus on positive and solving problem. 3. Predictors: Focus on positive, solving problem, and relaxing diversions. 4. Predictors: Focus on positive, solving problem, relaxing diversions, and ignore the problem. 5. Predictors: Focus on positive, solving problem, relaxing diversions, ignore the problem, and wishful thinking. 6. Predictors: Focus on positive, solving problem, relaxing diversions, ignore the problem, wishful thinking, and spiritual support.

**Table 7:** Regression model between self-esteem and the different coping strategies during treatment.

Model	R	R Square	R Square Change	Cumulative R Square	Sig.
1	0.551	0.304	0.304	0.304	<0.001
2	0.599	0.359	0.055	0.359	0.008
3	0.694	0.482	0.123	0.482	<0.001
4	0.746	0.556	0.075	0.557	<0.001
5	0.765	0.586	0.029	0.586	0.018

1. Predictors: Focus on positive. 2. Predictors: Focus on positive and ignore the problem. 3. Predictors: Focus on positive, ignore the problem, and wishful thinking. 4. Predictors: Focus on positive, ignore the problem, wishful thinking, and physical recreation. 5. Predictors: Focus on positive, ignore the problem, wishful thinking, physical recreation, and worry.

**Table 8:** Regression model between self-esteem and the different coping strategies during follow-up.

Model	R	R Square	R Square Change	Cumulative R Square	Sig.
1	0.595	0.353	0.353	0.353	<0.001
2	0.654	0.428	0.075	0.428	0.008
3	0.724	0.524	0.096	0.524	<0.001
4	0.753	0.566	0.042	0.566	<0.001

1. Predictors: Focus on positive. 2. Predictors: Focus on positive and ignore the problem. 3. Predictors: Focus on positive, ignore the problem, and wishful thinking. 4. Predictors: Focus on positive, ignore the problem, wishful thinking, and spiritual support.

**Table 9:** Regression model between self-esteem and different coping strategies during treatment in male adolescents.

Model	R	R Square	R Square Change	Cumulative R Square	Sig.
1	0.619	0.383	0.383	0.383	<0.001
2	0.751	0.564	0.182	0.565	0.008
3	0.812	0.659	0.095	0.660	<0.001
4	0.889	0.792	0.132	0.792	<0.001

1. Predictors: Keep to myself. 2. Predictors: Keep to myself and work hard. 3. Predictors: Keep to myself, work hard, and solving problem. 4. Predictors: Keep to myself, work hard, solving problem, and belong.

**Table 10:** Regression model between self-esteem and different coping strategies during treatment in female adolescents.

Model	R	R Square	R Square Change	Cumulative R Square	Sig.
1	0.633	0.400	0.400	0.400	<0.001
2	0.712	0.507	0.107	0.507	0.002
3	0.857	0.735	0.227	0.734	<0.001
4	0.889	0.790	0.056	0.790	0.001
5	0.916	0.838	0.048	0.838	<0.001

1. Predictors: Focus on positive. 2. Predictors: Focus on positive and ignore the problem. 3. Predictors: Focus on positive, ignore the problem, and wishful thinking. 4. Predictors: Focus on positive, ignore the problem, wishful thinking, and spiritual support. 5. Predictors: Focus on positive, ignore the problem, wishful thinking, spiritual support, and friends.

**Table 11:** Regression model between self-esteem and different coping strategies during follow-up in male adolescents.

Model	R	R Square	R Square Change	Cumulative R Square	Sig.
1	0.613	0.376	0.376	0.376	<0.001
2	0.719	0.518	0.141	0.517	0.001
3	0.841	0.708	0.190	0.707	<0.001
4	0.896	0.802	0.095	0.802	<0.001

1. Predictors: Not coping. 2. Predictors: Not coping and belong. 3. Predictors: Not coping, belong, and keep to myself. 4. Predictors: Not coping, belong, keep to myself, and ignore the problem.

**Table 12:** Regression model between self-esteem and different coping strategies during follow-up in female adolescents.

Model	R	R Square	R Square Change	Cumulative R Square	Sig.
1	0.722	0.521	0.521	0.521	<0.001
2	0.791	0.626	0.104	0.625	<0.001
3	0.836	0.699	0.074	0.699	<0.001
4	0.890	0.792	0.093	0.792	<0.001
5	0.920	0.846	0.054	0.846	<0.001

1. Predictors: Focus on positive. 2. Predictors: Focus on positive and physical recreation. 3. Predictors: Focus on positive, physical recreation, and ignore the problem. 4. Predictors: Focus on positive, physical recreation, ignore the problem, and keep to myself. 5. Predictors: Focus on positive, physical recreation, ignore the problem, keep to myself, and professional help.

## Data Availability

Data are contained within the article.
